# Cytogenomic characterization of small supernumerary marker chromosomes in patients with pigmentary mosaicism

**DOI:** 10.3389/fgene.2024.1356786

**Published:** 2024-04-16

**Authors:** M. P. Navarrete-Meneses, I. Ochoa-Mellado, R. Gutiérrez-Álvarez, D. Martínez-Anaya, U. Juárez-Figueroa, C. Durán-McKinster, E. Lieberman-Hernández, E. Yokoyama-Rebollar, S. Gómez-Carmona, V. Del Castillo-Ruiz, P. Pérez-Vera, C. Salas-Labadía

**Affiliations:** ^1^ Genetic and cancer Laboratory, National Institute of Pediatrics (Mexico), Mexico City, Mexico; ^2^ Genética Humana, Instituto Nacional de Pediatría, Mexico City, Mexico; ^3^ Laboratorio de Citogenética, Instituto Nacional de Pediatría, Mexico City, Mexico; ^4^ Departamento de Dermatología, Instituto Nacional de Pediatría, Mexico City, Mexico; ^5^ Departamento de Genética Médica, Centro de Rehabilitación e Inclusión Infantil Teletón, Cancún, México

**Keywords:** pigmentary mosaicism, small supernumerary marker chromosome (sSMC), cytogenomic analysis, fluorescence *in situ* cell hybridization (FISH), microarray

## Abstract

**Introduction:**

The combination of gene content on the marker chromosome, chromosomal origin, level of mosaicism, origin mechanism (chromothripsis), and uniparental disomy can influence the final characterization of sSMCs. Several chromosomal aberrations, including sSMCs, have been observed in 30%–60% of patients with pigmentary mosaicism, and in more than 80%, chromosomal abnormalities are present in the mosaic state. In patients with pigmentary mosaicism the most representative chromosomes involved in sSMCs are 3, 5, 6, 9, 10, 13, 15, 18, 20, and X. In this study, we included the complete clinical, cytogenetic, and molecular characterization of seven patients with pigmentary mosaicism associated with the presence of SMCs of different chromosomal origins.

**Methods:**

The patients were diagnosed by the Genetics and Dermatology Department of three different hospitals. Cytogenetic and FISH analyses were performed on peripheral blood, light skin, and dark skin. FISH analysis was performed using different probes, depending on the marker chromosome description. Different array analysis was performed.

**Results:**

To date, of the seven cases studied, the chromosomal origins of six were successfully identified by FISH or array analysis. The chromosomes involved in SMCs were 6, 9, 15, and 18, X. The most frequently found was the centric minute structure.

**Discussion:**

To date, this group of seven patients constitutes the largest clinical and cytogenetically finely described study of cases with pigmentary mosaicism associated with sSMCs. Undoubtedly, analysis of the two skin types is a fundamental part of our study, as numerical differences may occur in the cell lines found in each skin type. The knowledge generated in this study will help delineate a very heterogeneous entity more accurately, and in the future, analyzing more patients with PM will likely establish a more definite association with the presence of this genetic alteration.

## Introduction

Small supernumerary marker chromosomes (sSMCs) are defined as structurally abnormal chromosomes equal to or smaller than chromosome 20 of the same metaphase spread (Matsubara et al., 2020), with an incidence of 0.072%–0.075% in prenatal cases and 0.044% in newborns (Reddy et al., 2013; Sun et al., 2017; Pinto et al., 2018; Zhou et al., 2020; Marchina et al., 2021)**.** Notably, in patients with intellectual disabilities, the incidence may increase to 0.288% (Reddy et al., 2013; Zhou et al., 2020). Approximately 70% of sSMCs are *de novo*, and almost 70% are derived from acrocentric chromosomes, principally chromosome 15 (30%–50%) (Kraoua et al., 2011; Reddy et al., 2013; Pinto et al., 2018; Liehr and Al-rikabi, 2019; Matsubara et al., 2020; Zhou et al., 2020). sSMCs can have three different shapes and are described according to their frequency: inverted duplicated, centric minute, and ring-shaped sSMC (Liehr, 2012; Matsubara et al., 2020).

It has been suggested that partial trisomic rescue leads to the appearance of *de novo* sSMCs. This can be mediated by different events, such as U-type formation, ring chromosome formation, and chromothripsis (Kurtas et al., 2019; Liehr and Al-rikabi, 2019; Matsubara et al., 2020). As a consequence of incomplete trisomic rescue, the presence of mosaic with different cell lines is observed in approximately 50% of cases with sSMCs, with the mosaic of acrocentric chromosomes being the most frequently observed over non-acrocentric markers (Liehr T, 2013). As mentioned earlier, U-type exchange between different or within the same chromosome could leads to stable dicentric sSMCs, often acrocentric type mentioned as inverted duplicated (inv dup) sSMC (Ewers et al., 2010). In addition, in some cases, two copies of a chromosome of the same parent may be present in a normal cell, leading to uniparental disomy (UPD) (Liehr and Al-rikabi, 2019). Finally, the presence of sSMCs with neocentromeres, complex markers resulting from chromothripsis, and multiple sSMCs in the same individual must be considered (Tesner and Drabova, 2018)**.**

Frequently, with conventional cytogenetics, it is not possible to determine the chromosomal origin of sSMCs, so the use of molecular tools to characterize thzem accurately is very useful (Liehr, et al., 2011; Reddy et al., 2013; Xue et al., 2019; Li et al., 2020; Zhou et al., 2020). In addition, the genotype-phenotype correlation in patients with sSMC is challenging. The combination of gene content (presence of heterochromatin or euchromatin) on the marker chromosome, its chromosomal origin, level of mosaicism, origin mechanism (chromothripsis), and the presence of uniparental disomy can influence the final characterization of sSMC (Murthy et al., 2008; Liehr, 2013; Liehr, 2021; Marchina et al., 2021). The exceptions are a few syndromes with well-defined sSMC: isochromosomes (5p), (9p), (12p)/Pallister-Killian-syndrome, (18p), der (22)t(11; 22)/Emanuel syndrome, sSMC(15) containing the Prader-Willi/Angelman syndrome critical region, and sSMC(22), which contains the critical region for cat eye syndrome (Liehr T, 2013; Sun et al., 2017). Although sSMCs are considered rare, some entities such as pigmentary mosaicism (PM) show a high frequency of sSMCs, particularly in the mosaic state.

Pigmentary mosaicism (PM) is a heterogeneous group of skin pigmentation disorders characterized by the presence of hypopigmented and hyperpigmented macules, with or without extracutaneous manifestations. It is considered the third most common neurocutaneous condition, after neurofibromatosis type 1 and tuberous sclerosis (Kinsler, 2019). Several chromosomal aberrations have been observed in 30%–60% of PM cases, and in more than 80% of cases, chromosomal abnormalities are present in a mosaic state (Salas-Labadía et al., 2019). The presence of SMCs in PM patients has been reported previously and the most representative chromosomes involved are: 3, 5, 6, 9, 10, 13, 15, 18, 20, and X (Stanley et al., 1993; Boon et al., 1996; Portnoï et al., 1999; Hansen et al., 2003; Akahoshi et al., 2004; Lloveras et al., 2004; Gimelli et al., 2007; Villa, 2007; Murthy et al., 2008; Dhar et al., 2009; González-Enseñat et al., 2009; Baker et al., 2010; Oiso et al., 2010; Cappanera et al., 2011; Kraoua et al., 2011; Brock et al., 2012; Patil et al., 2012; Faletra et al., 2013; Myers et al., 2015; Cunha et al., 2016; Naoki et al., 2017; Samanta and Schaefer, 2020; Nisson et al., 2021)**.** However, the genotype-phenotype correlations of most sSMCs remain unknown and need to be established (Liehr et al., 2011). Thus, new information showing the clinical significance of sSMCs is relevant as it can have an impact on diagnosis and prognosis.

In this study, we included the complete clinical, cytogenetic, and molecular characterization of seven patients with pigmentary mosaicism associated with the presence of SMCs of different chromosomal origins.

## Material and methods

### Subjects

The patients were diagnosed by the Genetics and Dermatology Department of three different hospitals. This study was approved by the Research Ethics Committee of the National Commission of Bioethics, registration number “CONBIOETICA-09-CEI-025-20,161,215.” Signed informed consent was obtained in accordance with the recommendations of the Declaration of Helsinki.

### Cytogenetic analysis

Cytogenetic analysis was performed on peripheral blood (PB) lymphocytes following conventional techniques and interpreted according to the International System for Human Cytogenetic Nomenclature 2020 (ISCN) (McGowan-Jordan et al., 2020). Fresh biopsies were obtained from hypopigmented/Light skin (LS) and hyperpigmented/Dark skin (DS). Fibroblasts were cultured in complete Amniomax medium (Gibco) for 10–15 days. Cells seeded on glass coverslips were incubated with colcemid (10 mg/mL; Gibco, United States) for 20 min and harvested to obtain metaphase cells. G-banded metaphase cells were analyzed following the same criteria as for lymphocytes. Images were captured using an AXIO ImagerMI (Zeiss, Germany) microscope and IKAROS software (Meta Systems, Germany).

### FISH

FISH analysis with different probes was performed under the conditions recommended by the manufacturer (Abbot Molecular, Vysis, Downers Grove, IL, United States, and MetaSystems Probes). At least 15 metaphases were analyzed in each case.

### Molecular analysis

DNA was isolated from the peripheral blood for molecular studies using the Gentra^®^ PureGene^®^ Blood kit (Qiagen, Hilden, Germany) according to the manufacturer’s instructions. Chromosomal microarray analysis (CMA) was performed using aCGH 60 K (Agilent Technologies, Santa Clara, CA), (hg19 UCSC), CytoScan™ 750 K array, and SNP array (Thermo Fisher, United States or Illumina CRC BeadChip) (hg19/hg38) according to the manufacturer’s protocols. All data were visualized and analyzed using Chromosome Analysis Suite (ChAS 4.0) software (Thermo Fisher Scientific Inc.). The reporting threshold of the copy number result was set at 10 kbp with marker count ≥20 for gains, 10 kbp with marker count ≥20 for losses, and 3 Mb with marker count ≥50 for absence of heterozygosity (AOH). The analysis was based on the human reference genome version GRCh38 and interpreted with ClinVar (NCBI), DECIPHER, DGV, OMIM, ISCA, PubMed, ClinGen, and Genos Medica Laboratory databases.

## Results

### Patient 1

**FIGURE 1:**
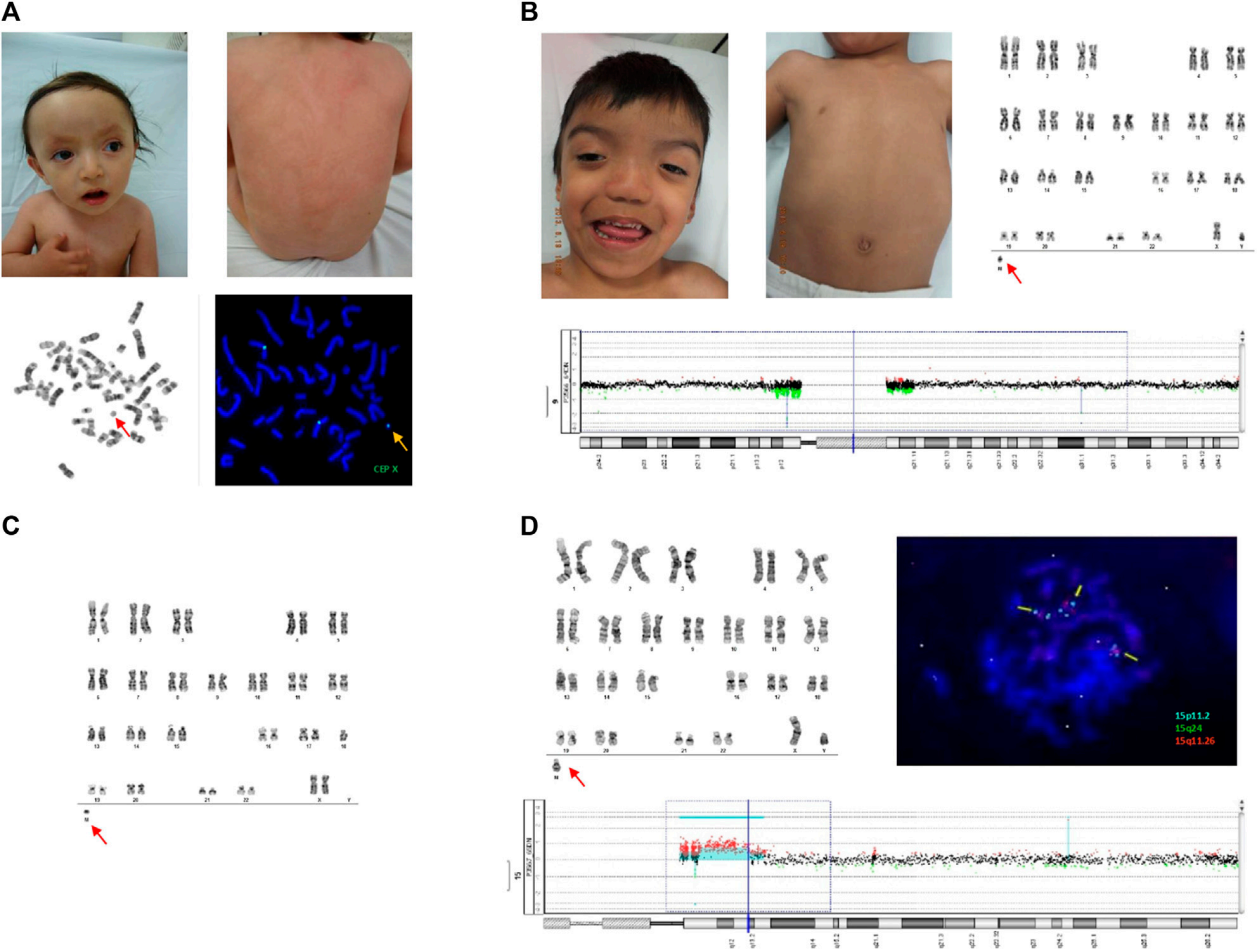
**(A)** Upper panels show dysmorphic facial features in patient 1 (left) and PM with fine BL in the dorsum (right). The lower panel shows G-banded metaphase, revealing the sSMC (left), and the FISH assay showing an sSMC CEP X positive signal (right). **(B)** Upper panels show dysmorphic facial features in patient 2 and PM with fine BL in the dorsum (left), and G-banded chromosomes with sSMC (right). Array CGH 60k is shown, revealing pericentromeric deletion of chromosome 9 (bottom). No information on sSMC was obtained using the array CGH. **(C)** G-banded chromosomes from patient 3 showing sSMC. **(D)** The upper panel shows the G-banded chromosomes of patient 4 with sSMC at the bottom (left). FISH assay with Vysis Prader-Willi/Angelman SNRPN probe showing sSMC with two aqua-positive and one orange-positive signals (right). aCGH 60k in the lower panel shows triple and quadruple doses in the 15q11 and 15q13 regions (Figure 1D; Table 1).

A 10-year-old female was referred for the first time at 1 year of age for West syndrome, pigmentary mosaicism (PM), and dysmorphic facial features (DFF) (Figure 1A). She was the first child of a non-consanguineous and healthy couple, with maternal and paternal ages of 24 and 29 years, respectively. Prenatal ultrasound revealed intrauterine growth retardation and oligohydramnios. She was delivered at 41 weeks by c-section because of fetal bradycardia. The birth weight was 3,200 g (z- 0.80 SD), length was 49 cm (z- 1.04 SD), and OFC was not available (SD were obtained based on Fenton growth charts according to gestational age). The Apgar score was 7/9 because of apnea. Psychomotor development was delayed (DD), with cephalic support at 7 months and sitting at 1 year. At 5 months of age, myoclonic seizures and spams were observed. Physical examination at 10 years 7-months-old showed high capillary implantation, bulging forehead, straight and spare eyebrows with a tendency of sinofris, proptosis, nasal tip down, and multiple nevi on her face. A short and broad neck, torax with teletelia, upper extremities with cubitus valgus, small hands, and a single transverse palmar crease were detected. Shortening of fourth and fifth metatarsal bones and the small fingers was observed in the lower extremities. In addition, disseminated dermatosis in the dorsum and lower limbs with multiple hypopigmented spots along the lines of Blaschko (BL) was observed (Figure 1A). Archetypical BL patterns are more likely to be of the 1A type (Kromann et al., 2018; Salas-Labadía et al., 2019). MRI showed bilateral frontal heterotopia, corticosubcortical atrophy, and hippocampal asymmetry.

Cytogenetic analysis revealed three different cell lines: one normal, other with a single “X”chromosome, and the other with a centric minute structure (min) sSMC (Liehr et al., 2011) in different proportions: PB: mos 47, XX,+mar [28]/45,X [6]/46, XX [16]; LS: mos 45,X [35]/47, XX,+mar [2]/46, XX [6]; DS: mos 45,X [20]/47, XX + mar [3]/46, XX [13] (Figure 1A; Table 1). To further characterize the sSMC, SNP array analysis (Illumina CRC BeadChip) was performed on DS with the following results: arr[GRCh37] Xp22.33q28(60,814–155,254,881)x1 [∼30%], Xp21.1p11.1(36,025,401–58,483,247)x3 [∼55%], Xp22.33q28(60,814–155,254,881)x2 [∼15%] (Human Genome Build 37, hg19) (Table 1). FISH analysis with an α-satellite X probe (CEP X) confirmed that the marker was a derivative of the X chromosome (Figure 1A; Table 1). Global analysis showed three different cell lines: mosaic with a monosomy X, duplication of a 22.46 Mb region within chromosome Xp21.1p11.1 (marker chromosome X), and normal cell lines.

**TABLE 1:** Overview of clinical manifestations and genetic findings.

Patient	Clinical manifestations	Cytogenetic analysis	Molecular analysis	Association with clinical manifestations
**Patient 1**	DD, DFF, and fine BL with disseminated hypo and hyperpigmentation in face, dorsum, and limbs	**PB:** mos 47, XX,+mar [28]/45,X [6]/46, XX [16]	**DS:** 45, X.arr[GRCh37] Xp22.33q28(60,814-155,254,881)x1 [∼30%], 47,XX,+mar.ish der(X)(DXZ1+). arr[GRCh37] Xp21.1p11.1(36,025,401-58,483,247)x3, [∼55%], 46,XX. ish der(X)(DXZ1+). arr[GRCh37] Xp22.33q28(60,814-155,254,881)x2 [∼15%]	**1)** Turner syndrome variant
		**LS:** mos 45,X [35]/47, XX,+mar [2]/46, XX [6]		**2)** Functional disomy Xp
		**DS:** mos 45,X [20]/47, XX + mar [3]/46, XX [13]		
**Patient 2**	DD, DFF, and camptodactyly. Fine BL with disseminated hypopigmented spots in lower limbs	**PB:** normal	**LS:** 47,XY, +mar.ish mar(D15Z1-,SNRPN-,PML-,D14/22Z1-,D13/21Z1-,D9Z1-). arr [GRCh37] 9p12p11.2(43,504,857-44,259,464)x1, 9q21.11(69,092,561-69,972,419)x1	No association with phenotype
		**LS:** mos 47, XY, 9qh-, +mar [19]/46, XY, 9qh- [33]		**1)** Heterochromatic constitution
		**DS:** mos 47, XY, 9qh-, +mar [9]/46, XY, 9qh- [42]		**2)** low-level mosaicism
				**3)** Neocentromere
**Patient 3**	DD, DFF, CHD, and seizures, fine BL with disseminated hypopigmented spots in dorsum and lower limbs	**PB:** normal	**LS:** 46, XX, -18, +mar.ish der(18)(D18Z1+). arr[GRCh38] 18p11.32p11.21(136,227_12,417,906)x1, 18q11.2q21.1(26,229,790_49,384,297)x1, 18q21.1q23(49,550,689_80,256,240)x1	**1)** 8.17 Mb region of 18q: critical region for cardiac abnormalities
		**LS:** 46, XX, -18, +mar [50]		**2)** *TMX3* associated with microphthalmia
		**DS:** mos 46, XX, -18, +mar [45]/46, XX [5]		
**Patient 4**	DD, DFF, low weight and length, and brachydactyly	**PB:** mos 47,XY,+mar [48]/46, XY [2]	**LS:** 47,XX,+mar.ish der(15)(D15Z1+,SNRPN+,PML-). arr[GRCh37] 15q11.1q11.2(20,394,272-22,759,378) x3, 15q11.2q13.2(22,759,378-30,509,325)x4, 15q13.2q13.3(30,509,325-32,706,883)x3	**1)** inv dup(15) syndrome
	Fine BL with disseminated hypopigmented spots in dorsum and inferior limbs	**LS:** mos 47,XY,+mar [46]/46, XY [4]		**2)** *CHRNA7* associated with DD, and poor speech
		**DS:** mos 47,XY,+mar [27]/46, XY [23]		**3)** P gene copy number changes related to abnormal skin pigmentation
**Patient 5**	DD, low weight and length, corpus callosum agenesis, pachygyria, bilateral hearing loss, and CHD. DFF, and BL with disseminated hypopigmented macules	**PB:** normal	**DS:** 47,XX,+mar.ish der(6)(IRF4x2).arr[GRCh38] 6p25.3p22.1(156974_28285082)x4	***1***) *FOXC1* associated with ocular development, segment dysgenesis and cardiac abnormalities
		**LS:** mos 47 XY, +mar [24]/46, XY [1]		**2)** *BMP6* associated with craniofacial abnormalities
		**DS:** mos 47,XY, +mar [23]/46, XY [2]		
**Patient 6**	Severe DD, DFF, CHD, and BL with disseminated hypopigmented spots in limbs	**PB:** mos 47,XY, +mar [18]/46, XY [7]	**LS:** 47,XX,+mar.ish der(9)(D9Z1+). arr[GRCh38] 9p21.2p13.2(25816220_37490580)x3	**1)** Phenotype associated with 9p21.2p13.2.9p duplication syndrome
		**LS:** mos 47,XY, +mar [13]/46, XY [12]		
*Patient 7	Minor DFF, microcephaly, and fine BL with disseminated hyperpigmented macules in lower limbs. Archetypical BL patterns 1A, C, and D	**PB:** mos 47,XX,+mar [22]/46, XX [3]	**PB: 47,XX,+mar.ish der(9)(wcp+,D9Z1+, 305J7-T7+)	**1)** tetrasomy 9p phenotype
				**2)** Mild phenotype associated with tetrasomy 9p only present in lymphocytes

**PB:** peripheral blood; **LS:** Light Skin (hypopigmented); **DS:** Dark Skin (hyperpigmented); **mos:** mosaic; []: number of cells with the specific chromosomal abnormalities; **BL:** blaschko lines; **DD:** developmental delay; **DFF:** dysmorphic facial features; **CHD:** congenital heart defect.

*In the mother of patient 7 we observed 0.06% of spontaneous chromatid breaks, and 0.08% of Gross Chromosomal Rearrangements: 3 cells with sSMCs (centric minute structure), and 1 cell with a translocation between chromosomes 1, and 7 (t(1; 7)(p21; q36) in 50 cells of PB, analyzed.

**wcp positive signal is associated with 24 Xcyte, and wcp 9 FISH.

### Patient 2

A 15-year-old male was referred for the first time at 4 months of age for persistent crying, postprandial vomiting, and DD. Twin pregnancy of a non-consanguineous couple with a mother of 29 and a father of 30 years old, respectively. He was delivered at 36 weeks by c-section because of preeclampsia. The birth weight was 2,055 g (z- 1.57 SD), length was 48 cm (z 0.34 SD), and OFC was 37 cm (z- 2.92 SD) (SD were obtained based on Fenton growth charts according to gestational age). The Apgar score was 8/9. Psychomotor development was delayed, with cephalic support at 4 months and no crawling at 1 year. Physical examination at 1 year revealed a narrow forehead, straight eyebrows, slightly down-slanting palpebral fissures, and telecanthus. Depressed and broad nasal bridge, hypoplastic nostrils, short columella, high and narrow palate, prominent chin, and cupped ears (Figure 1B). He presented with camptodactyly of the upper extremities. In addition, pigmentary mosaicism with disseminated hypopigmented spots and well-defined margins following fine Blaschko lines was observed (Figure 1B). Archetypical BL patterns are more likely to be of the 1A type (Kromann et al., 2018; Salas-Labadía et al., 2019). MRI showed hypoxic-ischemic periventricular leukomalacia.

Cytogenetic analysis revealed two different fibroblast cell lines: one normal cell line and one with a centric minute structure (min) sSMC (Liehr et al., 2011) in different proportions, as follows: LS: mos 47, XY, 9qh-, +mar [19]/46, XY, 9qh- [33]; DS: mos 47, XY, 9qh-, +mar [9]/46, XY, 9qh- [42] (Figure 1B; Table 1). PB was normal. Array analysis of LS (aCGH 60k) and FISH with different probes, including the Prader-Willi/Angelman SNRPN and α satellite probes 9, 14/22, and 13/21, did not show positive results for sSMC characterization (Table 1).

### Patient 3

A 3-year-old female was referred at 1 year 4 months for epilepsy, cardiopathy, developmental delay, microphthalmia of the right eye, ectasia and hypoplasia of the right kidney, and dermatosis. She was the first child of a non-consanguineous couple, with mother of 20 years and father of 21 years old. The patient was delivered at 41 weeks of gestation, with aspiration of the meconium that required hospitalization for 23 days. The birth weight was 2,750 g (z-1.83 SD), length was 49 cm (z-1.04 SD), and OFC was not available (SD were obtained based on Fenton growth charts according to gestational age). The Apgar score was 7/9. Psychomotor development was delayed with cephalic support at 5 months. At 2 years and 6 months, brachycephaly, a narrow forehead, spare eyebrows, ptosis, and right microphthalmia were observed. A depressed nasal bridge, dysplastic right ear, short neck, teletelia, and inverted nipples were also detected. She presented with congenital heart defects (CHD) and disseminated dermatosis (dorsum and lower limbs) consisting of multiple linear hypopigmented spots along the Blaschko lines. An archetypical BL pattern type 1A has been detected (Kromann et al., 2018; Salas-Labadía et al., 2019).

Cytogenetic analysis revealed a cell line with a centric minute structure (min) sSMC (Liehr et al., 2011) in different proportions, with the following results: LS: 46, XX, −18, +mar [50]; DS: mos 46, XX, −18, +mar [45]/46, XX [5] (Figure 1C; Table 1). PB was normal. Cytoscan 750k analysis (ThermoFisher, USA/hg38) was performed on LS with the following result: arr[GRCh38] 18p11.32p11.21(136,227_12,417,906)x1, 18q11.2q21.1(26,229,790_49,384,297)x1, 18q21.1q23(49,550,689_80,256,240)x1. FISH analysis with the α-satellite 18 probe (D18Z1+) showed a positive signal, confirming that the marker was a derivative chromosome 18. A negative result for the whole-chromosome painting probe for chromosome 18 (WCP) was obtained (data not shown). Global analysis showed LS with a mosaic of monosomy 18 in conjunction with a non-supernumerary marker chromosome 18, and DS with a cell line of monosomy 18 in conjunction with a non-supernumerary marker 18, and a normal cell line (Table 1). It is important to note that the Cytoscan 750k analysis showed the monosomy 18 cell line but did not reveal the cell line with the marker chromosome.

### Patient 4

A 12-year-old male was referred at 5 years old for language delay and short stature was referred to our hospital at 5 years of age. He was the second child of a non-consanguineous and healthy couple, with maternal and paternal ages of 41 and 38 years, respectively. Positive for consanguinity between parents. He was delivered at 39 weeks after a c-section without complications. The birth weight was 3,000 g (z- 0.80 SD), length was 50 cm (z- 0.10 SD), and OFC was not available (SD were obtained based on Fenton growth charts according to gestational age). The Apgar score was 9. Psychomotor development was delayed with cephalic support at 6 months, sitting with support at 10 months, sitting alone at 12 months, and walking alone at 20 months. At 6 years of age, the patient showed low weight (z-2.11), short length (z-2.95), arched eyebrows, downslating palpebral fissures, reverse epicanthus, tubular nose, and low-set-cupped ears. He presented with bifid uvula, torax with teletelia, a single transverse palmar crease on the right hand, and brachydactyly. Disseminated dermatosis, consisting of multiple hypopigmented spots along the Blaschko lines, was observed.

Cytogenetic analysis revealed the following results: LS: mos 47,XY,+mar [46]/46, XY [4]; DS: mos 47,XY,+mar [27]/46, XY [23]; and PB: mos 47,XY,+mar [48]/46, XY [2] (Figure 1D; Table 1). aCGH 60k analysis revealed the following result: arr 15q11.1q11.2(20,394,272–22,759,378)x3, 15q11.2q13.2(22,759,378–30,509,325)x4,15q13.2q13.3(30,509,325–32,706,883)x3 (Figure 1D; Table 1). FISH analysis with the Vysis Prader-Willi/Angelman SNRPN probe showed two aqua signals on 15p11.2 and one orange signal on 15q11.2, confirming an inverted duplicated sSMC shape (Figure 1D; Table 1) (Liehr et al., 2011). The karyotypes of both parents were normal.

### Patient 5

**FIGURE 2:**
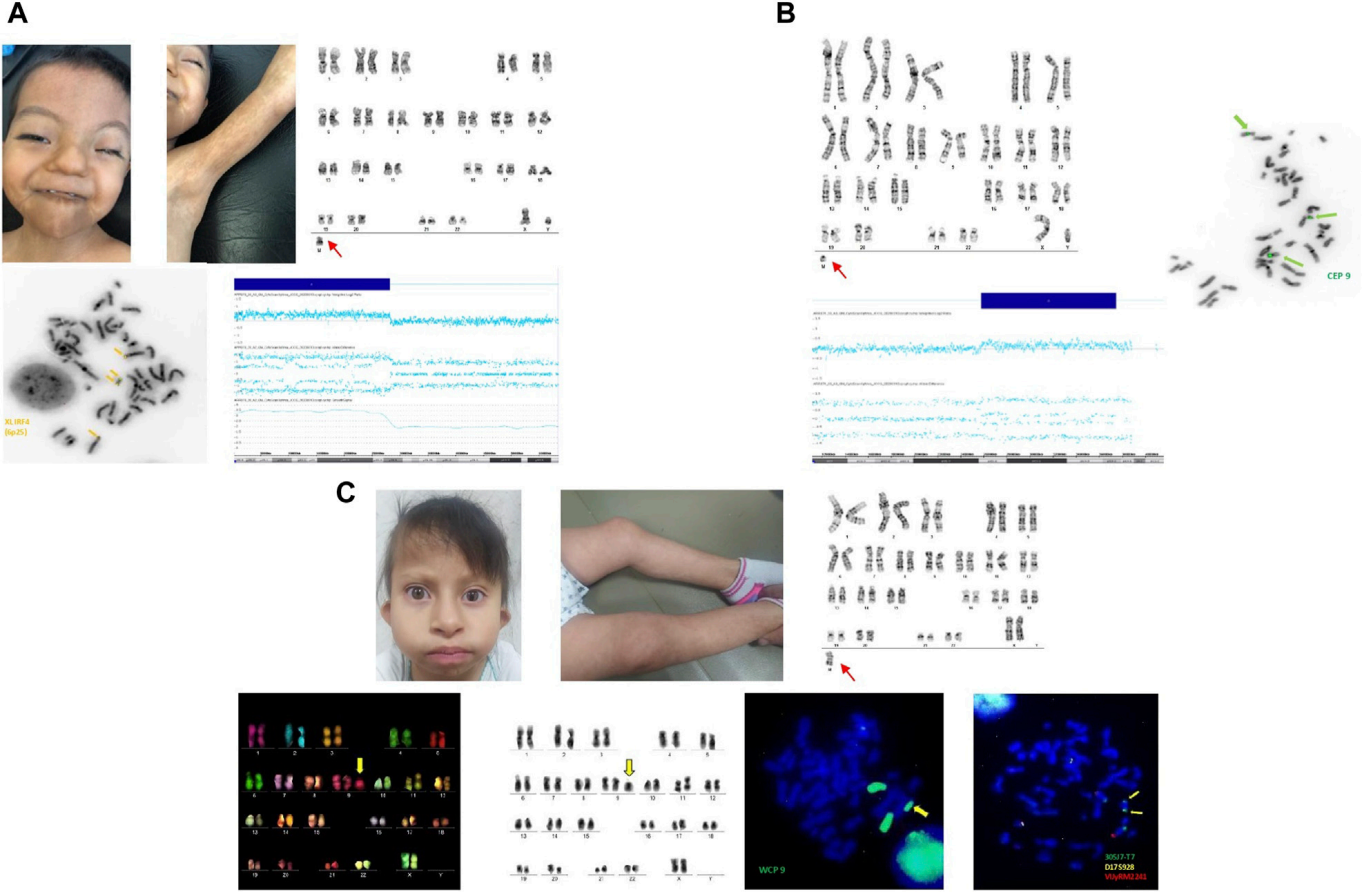
**(A)** Upper panels show dysmorphic facial features of patient 5 and and PM with fine BL in upper limbs (left). G-banded chromosomes showing sSMC (right). The lower panels show (left) the sSMC FISH assay with XL IRF4 (6p25) break-apart positive double signals, and the SNP-Array 46 Optima showing mosaicism of 6p tetraploidy on the right. **(B)** G-banded chromosomes of patient 6 with sSMC (left) and metaphase of FISH assay (right) showing a CEP 9 positive signal. SNP-Array 46 Optima revealing mosaic trisomy 9p (lower panel). **(C)** Dysmorphic facial features of patient 7 and PM with fine BL in the lower limbs (left). G-banded chromosomes showing sSMC at the bottom (right). FISH images are included, chromosomes of M-FISH assay revealing the nine chromosome origins of the sSMC, and chromosomes in inverted gray are shown in the lower panels (left). sSMC with a WCP nine positive signal, and two subtelomeric 9p positive signals, revealing a tetrasomy of 9p.

A 2-year-old male was referred at 1 year 11 months for pigmentary mosaicism and DFF. He was the first child of a non-consanguineous and healthy couple, with maternal and paternal ages of 29 and 35 years, respectively. He was delivered at 34 weeks of gestation by c-section because of ultrasound findings and oligohydramnios. The birth weight was 2,100 g (z- 0.38 SD), length was 49 cm (z 1.70 SD), and OFC was not available (SD were obtained based on Fenton growth charts according to gestational age). The Apgar score was 7/9. Delayed psychomotor development was detected without cephalic support and babbling at 1 year and 8 months. Low weight and length, corpus callosum agenesis, pachygyria, bilateral hearing loss, and congenital heart defects were also observed. Physical examination revealed a narrow forehead, upslanting palpebral fissures, hemifacial microsomia, broad nose, dysplastic ears, short neck, broad thorax, and cryptorchidism (Figure 2A). Pigmentary mosaicism with disseminated hypopigmented macules following the Blaschko lines was observed (Figure 2A). An archetypical BL pattern type 1A has been reported (Kromann et al., 2018; Salas-Labadía et al., 2019).

Cytogenetic analysis revealed two different cell lines: one normal and another with an sSMC similar to a centric minute structure (min) (Liehr et al., 2011) in different proportions: LS: mos 47 XY, +mar [24]/46, XY [1]; DS: mos 47,XY, +mar [23]/46, XY [2] (Figure 2A; Table 1). PB was normal. Cytoscan 750k revealed the sSMC origin as follows: arr[GRCh38] 6p25.3p22.1(156974_28285082)x4 (Figure 2A; Table 1). FISH analysis using the XL IRF4 break-apart probe (MetaSystems) allowed us to corroborate the shape of the sSMC as an inverted duplicated structure with two yellow signals (Figure 2A; Table 1).

### Patient 6

A 10-year-old boy was referred to our hospital with tonic seizures at 6 years of age. He was the second child of a mother aged 38 years and father aged 35 years, non-consanguineous. He was delivered at 41 weeks via c-section with a nuchal cord. The birth weight was 2,300 g (z- 3.45 SD), length was 52 cm (z- 0.03 SD), and OFC was not available (SD were obtained based on Fenton growth charts according to gestational age). The patient had a history of congenital cardiomyopathy. Severe psychomotor development was delayed with cephalic support at 2 years, sitting alone at 6 years, and no bladder control. Physical exploration at 6 years showed low weight (z-1.88), short length (z-2.81), developmental delay, microcephaly, and plagiocephaly. Arched eyebrows, upslanting palpebral fissures, thin superior lip, low-set and cupped ears, prominent antihelix, dextrocardia, thoracic asymmetry, and bilateral cryptorchidism were also observed. Aberrant palmar and plantar lines, finger pads, clinodactyly, and shortening of the second left toe were also detected. He presented with pigmentary mosaicism and disseminated dermatosis. Archetypical BL patterns are types 1A (Kromann et al., 2018; Salas-Labadía et al., 2019). Magnetic resonance imaging (MRI) revealed a wide cistern magna and cortico-subcortical atrophy.

Cytogenetic analysis revealed two different cell lines: one normal and another with an sSMC similar to a centric minute structure (min) (Liehr et al., 2011) in different proportions: LS: mos 47,XY, +mar [13]/46, XY [12]; PB: mos 47,XY, +mar [18]/46, XY [7] (Figure 2B; Table 1). The DS karyotype was not available. Cytoscan 750k analysis revealed the following results: arr[GRCh38] 9p21.2p13.2(25816220_37490580)x3 (Figure 2B; Table 1). FISH analysis using a centromeric probe for chromosome 9 (Abbot Molecular, Vysis, Downers Grove, IL, United States of America) corroborated these array results (Figure 2B; Table 1).

### Patient 7

A 4-year-old girl was referred to our hospital at 3 years of age because of pigmentary mosaicism. She was the first child of a non-consanguineous mother aged 20 years and a father of 40 years old. The birth weight was 2,350 g (z- 2.87 SD), length was 45 cm (z- 2.85 SD), and OFC was not available (SD were obtained based on Fenton growth charts according to gestational age). The Apgar score was 8/9. The patient presented with adequate psychomotor development. Physical examination revealed microcephaly, micrognathia, low-set ears, and disseminated dermatosis consisting of multiple hyperpigmented macules, followed by a phylloid pattern in the trunk, a checkerboard pattern in the buttocks, and fine Blaschko lines in the lower limbs (Figure 2C). Archetypical BL patterns are types 1A, C, and D (Kromann et al., 2018; Salas-Labadía et al., 2019).

Cytogenetic analysis revealed PB with two different cell lines: mos 47,XX,+mar [22]/46, XX [3] (Figure 2C; Table 1). LS and DS were normal. To characterize the marker chromosome, 24 Xcyte FISH (MetaSystems) was performed, which revealed the marker origin as a derivative of chromosome 9 (Figure 2C; Table 1). FISH with WCP 9, and subtelomeric 9p probes showed positive signals (Figure 2C; Table 1). Cytogenetic analysis was performed on both parents. Interestingly, the mother showed chromosomal instability in two independent samples of PB, principally chromatid break type (involving chromosomes 2,6,8), 3 cells with a “min” marker chromosome, and 1 cell with a translocation (t(1; 7)(p21; q6).

## Discussion

Small supernumerary marker chromosomes are still recognized as a challenge for clinical cytogenetics, given that their fine characterization requires not only traditional banding cytogenetics, but also molecular methods. Furthermore, the clinical significance of sSMCs is complicated when they exist in a mosaic state coexisting with normal cell lines. Approximately 60% of mosaic cases with sSMCs show clinical abnormalities; however, no correlations have been reported with the level of mosaicism in the peripheral blood (Liehr et al., 2006)**.** Thus, information showing associations between clinical abnormalities and mosaicism in sSMCs is very relevant, as it is recognized that this relationship is not simple. Chromosomal abnormalities are key to explaining such clinical variability as the pathogenic basis of PM. When both leukocytes and cultured fibroblasts were evaluated in the same patient, several chromosomal aberrations (including sSMC) were observed in 30%–60% of PM cases, and almost 80% of the cases were present in a mosaic state. Notably, it is assumed that the presence of differential skin pigmentation could be related to two distinct genotypes in each skin type (Salas-Labadía et al., 2019). Undoubtedly, analysis of the two skin types is a fundamental part of our study, as numerical differences may occur in the cell lines found in each skin type. Although more studies are required to obtain a conclusion suggesting the existence of an association between the presence of these markers and the presence of pigmentary alterations and other manifestations in other systems, the description of these seven patients contributes significantly to what has already been reported in these patients.

To date, this group of seven patients constitutes the largest clinical and cytogenetically finely described study of cases with pigmentary mosaicism associated with small supernumerary marker chromosomes. Following a strict cytogenetic analysis strategy, of a total population of 122 p.m. patients (73 patients was already reported in Salas-Labadía et al., 2019), we found that 36/122 (29.5%) patients with PM and other extracutaneous manifestations, had chromosomal abnormalities classified as follows: 1) patients with translocations X; autosomes or other translocations (n = 4); 2) patients with characterized ring chromosomes (n = 3); 3) patients with other structural abnormalities, some of them marker chromosomes already finely described and associated with the specific patient phenotype (n = 10); 4) patients with numerical alterations (n = 12) and 5) patients with sSMCs without characterization (n = 7). These seven last patients are the group included in this study, and it is important to mention that until now it is the largest population with PM associated to sSMCs described and characterized.

As a general rule, pigmentary abnormalities were the main inclusion criteria, with or without extracutaneous manifestations. Phenotype associations with marker chromosomes is challenging and depends on many factors, as mentioned previously. The patients described here showed great variability in terms of the chromosomal origin and shape of the marker as well as in terms of the tissues with their presence and the number of affected cells. All these factors contributed to the clinical heterogeneity observed in these patients.

### Patient 1

In patient 1, we found monosomy of chromosome X, duplication of Xp21.1p11.1, and a normal cell line. These results are consistent with 1) Turner syndrome variant. We found features associated with monosomy X, such as a short and broad neck, broad torax with teletelia, upper extremities with cubitus valgus, multiple nevi, and shortening of fourth and fifth metatarsal bones. 2) Functional disomy Xp. Occurs when the X chromosome inactivation center is separated from region Xp; because of this, the phenotype could be abnormal as a result of the expression of duplicated genes that are supposed to be silent (Sheath et al., 2013). Patients with functional disomy of similar or larger portions of Xp have been reported to have more severe phenotypes (Sheath et al., 2013). Other patients with functional disomy for a portion of the Xp overlapping with the patient’s findings have been reported to have developmental delay (DD) and dysmorphic facial features (DFF), as described in this patient (Kolomietz et al., 2005; Sheath et al., 2013). Notably, the Xp duplication associated with the sSMC contains a large number of genes, but not the XIST gene [OMIM # 314670], which codes for the X-inactivation center. We would expect this patient to have a functional disomy of this duplicated region, resulting in increased expression of the included genes. This increased expression may explain the more severe phenotype observed in this patient. Another report described duplication Xp11.2-p21.3, which shares similar clinical manifestations with our patient, including DD and cutaneous manifestations (Gustashaw et al., 1994). It is important to consider that genetic counseling should be recommended to parents (Baker et al., 2010; Kromann et al., 2018; Liehr, 2021; Liehr, 2021).

### Patient 2

Patient 2 showed phenotypical manifestations such as DD, DFF, and PM. In this patient, a genotype-phenotype correlation related to the presence of sSMC could not be obtained because the chromosomal origin could not be confirmed. However, it is emphasized the need to use different molecular tools to finally know its chromosomal origin. The negative results obtained with array analysis suggest a heterochromatic constitution or the need for better sensitivity to detect low-level mosaicism. The presence of a neocentromere and negative FISH results should be considered. It is important to note that the utilization of unfreeze fibroblasts cultures, could modify the percentage of cells with the alteration (Liehr, 2012; Liehr T, 2013; Xue et al., 2019). Finally, it is important to consider the presence of UPD as the causal origin of clinical manifestations in this patient (Liehr et al., 2011).

The array results in this patient showed a deletion in two regions of chromosome 9: 9p12-p11.2 (754,6 Kb) and 9q21.11 (879,9 Kb). Ivanov et al., 2018 reported a patient with a 9q21.11-q21.2 microdeletion of about 9.655 Mb in size. The clinical manifestations observed in our patient were similar to those seen in previously reported patients: developmental delay and some dysmorphic facial features, such as down-slanting palpebral fissures, broad nasal bridge, narrow and highly palatable, and prominent chin. Interestingly, pigmentary anomalies are only present in patient 2. At this point, some phenotypic features in our patient could be associated more with the microdeletion on chromosome 9, and the contribution of the marker to the phenotype remains unknown (Ivanov et al., 2018).

### Patient 3

We decided to include this patient in our description of sSMC associated with PM because although it is not a supernumerary marker, it is included within the “size” required for it and is associated with the clinical manifestations of interest. Could be considered as similar imbalances-no sSMC (Liehr, 2021).

Patients with 18p deletions have heterogeneous clinical manifestations, including hypotony (84%), MRI alterations (66%), otitis (61%), cardiomyopathy (56%), ptosis, and strabismus (55%) (Hasi-Zogaj et al., 2015; Jin et al., 2021). Patient 3 showed ptosis, short neck, kidney malformations, and cardiomyopathy. Of the 196 genes on 18q, only 15 (8%) were hemizygous, leading to haploinsufficiency and an abnormal phenotype. The other 10 genes are categorized as conditionally dosage-sensitive, so when they are hemizygous, they are a risk factor for an abnormal phenotype. The terminal 8.17 Mb region of 18q was identified as a critical region for cardiac abnormalities (Cody et al., 2015). The *TMX3* [OMIM#616102] gene, localized on chromosome 18q22.1, has been reported to be associated with microphthalmia (Chao et al., 2010). Pigmentary alterations, as well as CHD, have been reported in a patient with 2 cell lines, one with a deletion 18(q21q23) and one with a marker chromosome derived from chromosome 18 (Woods et al., 1994; Liehr, 2021).

In this patient, the sSMC was also identified as chromosome 18 using the D18Z1 probe; however, the array analysis did not help with marker characterization. This was probably due to the marker size, suggesting a heterochromatin constitution.

### Patient 4

Chromosome 15 sSMC account for 50% of the total sSMCs, of which 80% are present as an inverted duplication of 15 (Lu et al., 2020). The mechanism responsible of the 80% of the supernumerary chromosome 15 resulting in tetrasomy for 15q11.2- q13.1, are result from maternal isodicentric 15q11.2-q13.1 ((Lu et al., 2020). In most cases of inv dup(15) syndrome, only maternally inherited duplication or triplication is pathogenic and is associated with abnormal brain development (Chen et al., 2016). The inv dup sSMC found in the present case resulted in a shared tetrasomy of the *NDN, SNRPN, and UBE3A* genes, and trisomy of *CHRFAM7A* and *CHRNA7* genes associated with DD, and poor speech; both are clinical manifestations reported in this patient (Chen et al., 2016; Chilakamarri and Mellin-, 2022). Minor DFF-like downslating palpebral fissures, low-set-cupped ears, and brachydactyly have been previously reported and are present in our patient. Changes in skin pigmentation have been reported occasionally (Akahoshi et al., 2004; Battaglia, 2008; Rosado et al., 2012; Li et al., 2018; Liehr, 2021).

### Patient 5

Tetrasomy 6p is considered extremely rare. To our knowledge, this is the third reported case of partial tetrasomy of the distal 6p. The first reported patient showed tetrasomy 6p not associated with sSMCs (Ryan et al., 2007), and the second reported case was very similar to our patient, who showed an inverted duplicated sSMC from 6p (Syu et al., 2022)**.** Mosaic tetrasomy of the distal 6p caused by sSMC was associated with multisystem malformations, including craniofacial, cardiovascular, genitourinary, ophthalmological, and hearing problems, which was attributed to a duplication of 14.337 Mb in the 6p23-p25.3 region, which involved 65 genes (Syu et al., 2022). Our patient shares many of these clinical manifestations, including craniofacial, cardiovascular, genitourinary, and hearing defects. Interestingly, renal defects, which were seen in trisomy 6p cases and were found in tetrasomy 6p patients, were not detected in our patient. The only genitourinary feature observed in this patient was cryptorchidism. In addition, PM has not previously been reported in patients with trisomy or tetrasomy 6p. Our patient showed a larger duplicated region than the case reported by Syu et al., 2022; the 6p25.3p22.1 region duplicated in our patient involved 274 genes. Among the affected genes, *FOXC1* and *BMP6* were detected, which have been suggested to contribute to the 6p tetrasomy phenotype. FOXC1 is located at 6p25.3 and play a role in embryonic and ocular development; it has been related to segment dysgenesis and cardiac abnormalities. Although *FOXC1* has also been associated with kidney development, renal defects were not observed in this patient. *BMP6* is located at 6p24.3, and its overexpression is associated with craniofacial abnormalities (Liehr, 2021; Villa, 2007; Syu et al., 2022).

### Patient 6

9p21.2p13.2.9p duplication syndrome is characterized by short stature, DD, intellectual disability, and DFF (bulbous nose tip, hypertelorism, deep-set eyes, down-turned corners of the mouth, low-set ears, and short neck) (Tkemaladze et al., 2023). Clinical manifestations in our patient were consistent with findings reported in the literature: short stature, DD, up-slanting palpebral fissures, and low-set ears. Guilherme et al., 2014 reported a patient with a 9p21.2 duplication and cryptorchidism, also present in this patient (Guilherme et al., 2014). Patients with pigmentary mosaicism and duplication of 9p have not been reported (Liehr T et al.; Patil et al., 2012; Sams et al., 2022).

### Patient 7

Cytogenetic analysis revealed an extra chromosome of unknown origin, despite being a marker that did not correspond in size to chromosome 20. We decided to include it in our study because of the unknown origin of the SMC and because it was associated with the presence of pigmentary mosaicism. Although G-banding suggested chromosome 9 as the origin of the marker chromosome, we could only confirm this through M-FISH. Performing an additional FISH assay with subtelomeric probes on chromosome nine allowed us to confirm the tetrasomy of 9p.

Tetrasomy 9p is a rare chromosomal abnormality that presents as an isochromosome or isodicentric chromosome derived from the short arm of chromosome 9 (El Khattabi et al., 2015). To date, 68 cases of tetrasomy 9p have been reported (Kok Kilic et al., 2022) including 22 prenatal cases. Patients with tetrasomy 9p show variable phenotypic features. Although the phenotypic variability of this condition is unknown, it is suggested that it depends on the degree of mosaicism, if it is present in a single tissue or in several tissues, and also depends on the regions of chromosome nine involved. It has been suggested that the severity of clinical features increases if regions of 9q are involved, and it has also been reported that patients with i(9p) in fibroblasts tend to have more severe phenotypes than those whose isochromosomes are limited to lymphocytes, especially in terms of cardiac defects and viability (El Khattabi et al., 2015). The tetrasomy 9p phenotype ranges from multiple congenital anomalies with severe intellectual disability and growth delay, to subnormal mental and physical development. Hypertelorism or telecanthus, abnormally formed, low-set ears, microretrognathia, and a bulbous nose are the most common dysmorphic traits. Several cases of microcephaly, growth retardation, joint dislocation, scoliosis, cardiac defects, and renal anomalies have been reported. Additionally, PM has been reported in patients with tetrasomy 9p. These physical anomalies are often accompanied by intellectual disabilities, but not universally (Lloveras et al., 2004; El Khattabi et al., 2015; Liehr, 2021). Our patient showed some of these clinical features including microcephaly, micrognathia, low-set ears, and PM. No cardiac or renal defects were found in our patient; a mild phenotype was expected because tetrasomy 9p was exclusively found in lymphocytes. We could not further characterize the sSMC; thus, we cannot rule out the possibility that the marker chromosome contains 9q sequences. Further characterization of these markers will be performed.

Interestingly, structural chromosomal abnormalities (SCA), including chromatid breaks and other chromosomal rearrangements (sSMCs and t(1; 7)(p21; q36)) in two independent PB cultures, were described in the mother of patient 7. In total, 14% of SCA was observed in the 50 cells analyzed. In a previous study analyzing PB in healthy individuals, it was reported 0.06% of SCA included chromosomal and chromatid breaks and GCR (or rejoined rearrangements). With this we can observe a real tendency for chromosomal instability in the mother (Salas C et al., 2012). Finally, we suggested as the mechanism of marker chromosome origin a U-type exchange during meiosis (Liehr et al., 2004).

## Conclusion

The strict criteria for screening to realize the cytogenetic study permitted us to detect patients with sSMCs and discard the presence of mosaicism in two different tissues, PB and skin fibroblasts of light and dark skin (hypo and hyperpigmented). Therefore, it was of great interest to obtain the best characterization of the sSMCs in cases in which they could not be identified due to cytogenetics. The challenge of characterizing sSMCs of unknown origin has led us to use one or more molecular methodologies to accurately delineate these chromosomes and guides the patient’s diagnosis. In patients with marker chromosomes the association with phenotype is challenging and depends on many factors, as mentioned previously. In addition, the patients described here showed great clinical and genetic variability in terms of the chromosomal origin and shape of the marker, as well as in terms of the tissues with their presence and the number of affected cells. The knowledge generated in this study will help delineate a very heterogeneous entity more accurately, and in the future, analyzing more patients with PM will likely establish a more definite association with the presence of this genetic alteration.

The study of these seven patients undoubtedly generates knowledge that enriches the subsequent identification and characterization of marker chromosomes in entities as common as already established syndromes, as well as rare diseases, within which our entity is included. Until now, the cases described here together with those already reported in our population with chromosomal markers (finely described and associated with PM patient’s phenotype), and those reported in the literature, probably help us to be able to associate the clinical manifestations including PM with these chromosomal alterations.

## Data Availability

The datasets for this article are not publicly available due to concerns regarding participant/patient anonymity. Requests to access the datasets should be directed to the corresponding authors.
